# P73 tumor suppressor and its targets, p21 and PUMA, are required for madin-darby canine kidney cell morphogenesis by maintaining an appropriate level of epithelial to mesenchymal transition

**DOI:** 10.18632/oncotarget.4374

**Published:** 2015-06-08

**Authors:** Yanhong Zhang, Ashley Young, Jin Zhang, Xinbin Chen

**Affiliations:** ^1^ Comparative Oncology Laboratory, Schools of Medicine and Veterinary Medicine, University of Californian at Davis, Davis, CA, USA

**Keywords:** P73, p21, PUMA, epithelial-to-mesenchymal transition (EMT), MDCK

## Abstract

P73, a member of p53 tumor suppressor family, plays a crucial role in tumor suppression and neural development. Due to the usage of two promoters, p73 is expressed as two isoforms, TAp73 and ΔNp73, with opposing functions. Here, we investigated the potential role of p73 in epithelial polarity and morphogenesis by using Madin-Darby canine kidney (MDCK) cells as a model system. We found that knockdown of TAp73 enhances, whereas knockdown of ΔNp73 inhibits, MDCK cell proliferation and migration in two-dimensional (2-D) culture. We also found that knockdown of TAp73, but not ΔNp73, disrupts cyst formation of MDCK cells in three-dimensional (3-D) culture. Interestingly, we found that p21 and PUMA, both of which are induced by TAp73 but repressed by ΔNp73, are required for suppressing cell proliferation and migration in 2-D culture and for MDCK ce ll morphogenesis in 3-D culture. Finally, we showed knockdown of TAp73, p21 or PUMA induces epithelial to mesenchymal transition (EMT) with a decrease in E-cadherin and an increase in EMT transcription factors. Together, our data suggest that TAp73, p21 and PUMA play a critical role in modulating MDCK cell morphogenesis by maintaining an appropriate level of the EMT.

## INTRODUCTION

p73 belongs to the p53 family, which also includes p53 and p63. All three p53 family members are transcriptional factors and share a high sequence similarity, especially in the DNA binding domain. Interestingly, p73 is expressed as multiple isoforms, called TAp73 and ΔNp73, with opposing functions. TAp73 is transcribed from the upstream P1 promoter and contains a transactivation domain, similar to the one in p53. ΔNp73 is transcribed from the downstream P2 promoter in intron 3, which lacks the N-terminal transactivation domain. Consequently, TAp73 can induce cell cycle arrest or apoptosis and are considered as a bona fide tumor suppressor like p53 [1, 2]. By contrast, ΔNp73 is thought to be transcriptionally inactive and has oncogenic potential by antagonizing p53 or TAp73. In support of this notion, *in vivo* studies using mouse models showed a different role for TAp73 and ΔNp73 in tumor suppression and neuronal development. Specifically, mice deficient in TAp73 exhibit an increased incidence of both spontaneous and 7,12-dimethylbenz [α] anthracene (DMBA)-induced tumors [3], and accelerated aging [4]. Conversely, mice deficient in ΔNp73 do not develop tumors but are prone to delayed onset of moderate neurodegeneration, which largely phenocopied total p73 knockout mice [5, 6]. However, the underlying mechanism by which p73 controls tumor suppression and development is still uncertain.

Cyst formation, a key event for tubulogenesis during kidney development, involves cell polarization, proliferation, differentiation, apoptosis, cell-cell adhesion, migration and invasion [7, 8]. Recently, three-dimensional (3-D) cell culture models have allowed researchers to gain more mechanistic insights into the epithelial architecture morphogenesis. For example, MDCK cells can form polarized cystic structure, which can be induced to form branching tubules with lumens upon stimulation by hepatocyte growth factor (HGF) in 3-D culture [9, 10]. This process recapitulates many of the physiological characteristics of lumen formation during epithelial development and shares many morphological similarities to an epithelial tissue [11]. Interestingly, we showed previously that both p63 and mutant p53 play a role in regulating MDCK morphogenesis [12, 13]. However, it is not clear whether p73 is involved in this process.

In the current study, we explored the role of p73 in regulating MDCK morphogenesis in 2-D and 3-D cultures. Specifically, we found that stably knockdown of TAp73, but not ΔNp73, in MDCK cells enhances cell proliferation and migration in 2-D cultures and disrupts regular cyst structure in 3-D cultures. Similarly, we found that p21 and PUMA, both of which are TAp73 downstream targets, are required for maintaining MDCK morphogenesis. Furthermore, we showed that TAp73, p21, and PUMA regulate MDCK morphogenesis at least in part by maintaining an appropriate level of epithelial-to-mesenchymal transition (EMT).

## RESULTS

### Knockdown of TAp73 alters the morphogenesis of MDCK cells in 2-D and 3-D cultures

**Figure 1:**
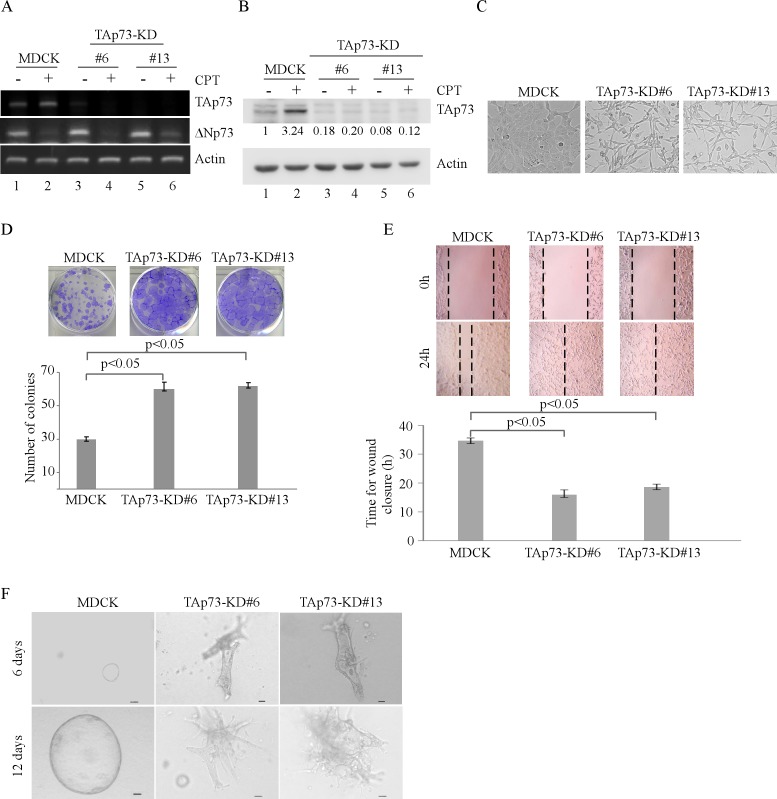
Knockdown of TAp73 alters the morphogenesis of MDCK cells in 2-D and 3-D cultures **A.** Generation of MDCK cell lines in which TAp73 was stably knocked down. The levels of TAp73, ΔNp73 and actin transcripts were determined in parental MDCK and MDCK-TAp73-KD cells by RT-PCR. **B.** Parental MDCK and MDCK-TAp73-KD cells were mock-treated or treated with camptothecin (CPT) for 18 h and the levels of TAp73 and actin proteins were determined by Western blotting with antibodies against TAp73 and actin. The relative level of TAp73 was normalized to that of actin and arbitrary set as 1.0 in parental MDCK cells. The relative fold change in the level of TAp73 protein was shown below each lane. **C.** Representative microscopic images of parental MDCK and MDCK-TAp73-KD cells in 2-D cultures. **D.** Top panel: colony formation assay was performed with MDCK or MDCK-TAp73-KD cells. Bottom panel: the number of colonies was counted and presented as Mean ± s.d. from three independent experiments. **E.** Wound healing assay was performed with MDCK or MDCK-TAp73-KD cells. Top panel: cell migration was determined by visual assessment of cells migrating into the wound for 24 h using a phase-contrast microscopy. The dashed lines indicate the wound edge. Bottom panel: the time required for would closure was measured and presented as Mean ± s.d. from three independent experiments. **F.** Representative images of MDCK or MDCK-TAp73-KD cells in 3-D culture for 6 d or 12 d. Scale bar: 50 μM

To determine the role of TAp73 in cell morphogenesis, MDCK cell lines in which TAp73 was stably knocked down were generated. Two representative clones, #6 and #13, were shown in Figure 1A-1B. We showed that upon treatment with camptothecin (CPT), TAp73 transcription was increased (Figure 1A, lanes 1-2), consistent with previous reports [14, 15]. We also showed that both TAp73 mRNA and protein levels were significantly reduced in TAp73-KD cells as compared to that in parental MDCK cells regardless of CPT treatment (Figure 1A-1B). In addition, we showed that the level of ΔNp73 transcript was slightly increased by TAp73 knockdown and can be inhibited by CPT treatment (Figure 1A, ΔNp73 panel), consistent with previous report [16]. We would like to mention that due to the low reactivity of ΔNp73 antibody, we were unable to detect endogenous ΔNp73 protein in MDCK cells. Interestingly, we found that unlike parental MDCK cells, MDCK-TAp73-KD cells exhibited spindle-shaped morphology in 2-D culture, which represents the property of mesenchymal cells (Figure 1C). Moreover, we found that knockdown of TAp73 promoted MDCK cell proliferation (Figure 1D) and migration (Figure 1E) in 2-D culture. Next, we determined whether knockdown of TAp73 affects MDCK cell polarity and morphogenesis in 3-D culture. To address this, both parental MDCK and MDCK-TAp73-KD cells were cultured in 3-D collagen gel for 6–12 days. As expected, parental MDCK cells formed a polarized cyst structure (Figure 1F, left panels), consistent with previous report [12]. By contrast, MDCK-TAp73-KD cells lost their polarity and formed irregular cyst structures (Figure 1F, right two panels). Together, these data suggest that knockdown of TAp73 in MDCK cells promotes cell growth and migration in 2-D cultures and disrupts cyst formation in 3-D cultures.

### Knockdown of ΔNp73 suppresses MDCK cell proliferation and migration in 2-D culture and delays cyst formation in 3-D culture

**Figure 2:**
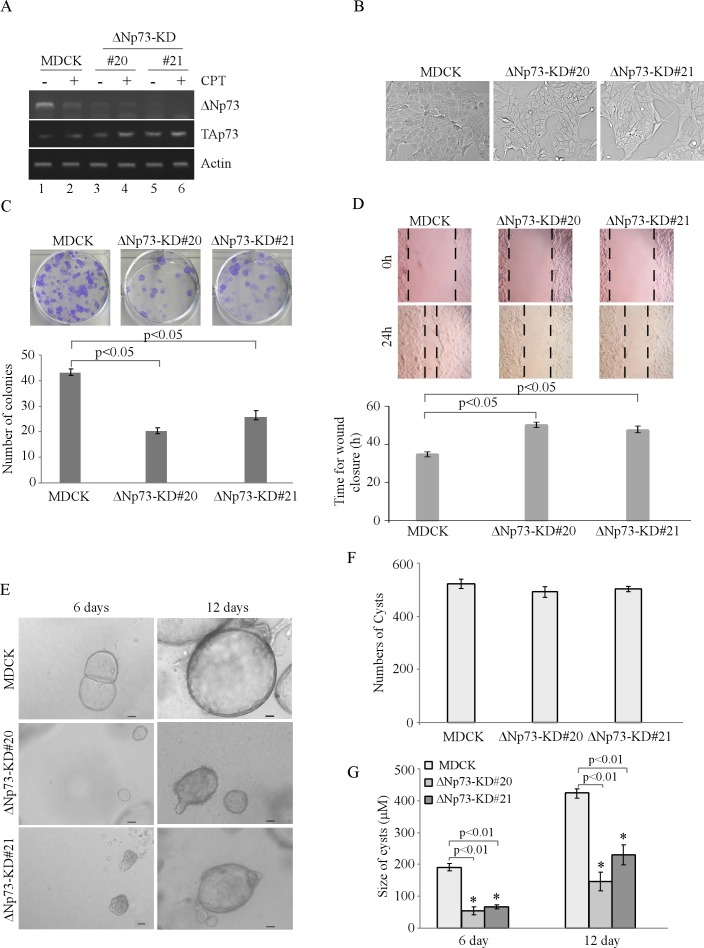
Knockdown of ΔNp73 in MDCK cells suppresses cell proliferation and migration in 2-D culture, and delays cyst formation in 3-D culture **A.** Generation of MDCK cell lines in which ΔNp73 was stably knocked down. The levels of ΔNp73 and TAp73 transcripts were determined in parental MDCK and MDCK-ΔNp73-KD cells by RT-PCR. **B.** Representative images of parental MDCK and MDCK-ΔNp73-KD cells in 2-D culture. **C.** Top panel: colony formation assay was performed with parental MDCK and MDCK-ΔNp73-KD cells. Bottom panel: the number of colonies was counted and presented as Mean ± s.d. from three separate experiments. **D.** Wound healing assay was performed with parental MDCK and MDCK-ΔNp73-KD cells. Top panel: cell migration was determined by visual assessment of cells migrating into the wound for 24 h using a phase-contrast microscopy. The dashed lines indicate the wound edge. Bottom panel: the time required for would closure was measured and presented as Mean ± s.d. from three separate experiments. **E.** Representative images of parental MDCK and MDCK-ΔNp73-KD cells in 3-D culture for 6 d or 12 d. Scale bar: 50μM. **F.** Cyst number was counted at day 12 in 3-D culture in MDCK or MDCK-ΔNp73-KD cells and present as Mean ± s.d. from three separate experiments. **G.** The size of cysts in 3-D culture was measured under microscope and presented as Mean ± s.d. from three separate experiments. * indicated as *p* < 0.01 (by student *t*-test).

To determine the role of ΔNp73 in cell morphogenesis, MDCK cell lines in which ΔNp73 was stably knocked down were generated and two representative clones (clones #20 and #21) were shown in Figure 2A. We showed that the level of ΔNp73 transcript was almost undetectable in both MDCK-ΔNp73-KD cells regardless of CPT treatment (Figure 2A, ΔNp73 panel). In addition, we showed that knockdown of ΔNp73 had little effect on TAp73 transcripts, which can still be up-regulated by CPT treatment (Figure 2A, TAp73 panel). Notably, unlike MDCK-TAp73-KD cells, the morphology of MDCK-ΔNp73-KD cells was very similar to that of parental MDCK cells (Figure 2B). Moreover, knockdown of ΔNp73 inhibited MDCK cell proliferation and migration in 2-D culture (Figure 2C-2D). Furthermore, we found that knockdown of ΔNp73 in MDCK cells led to smaller cyst formation in 3-D culture (Figure 2E). Thus, we quantitated the cyst number and size of both parental MDCK and MDCK-ΔNp73-KD cells. We found that knockdown of ΔNp73 did not decrease the number but significantly reduced the size of cysts in 3-D culture (Figure 2F-2G). Together, these data suggest that ΔNp73 is required for MDCK cell proliferation and migration in 2-D culture, delays but not disrupts cyst formation in 3-D culture.

### p21 and PUMA are required for MDCK cell morphogenesis in 2-D and 3-D cultures

**Figure 3:**
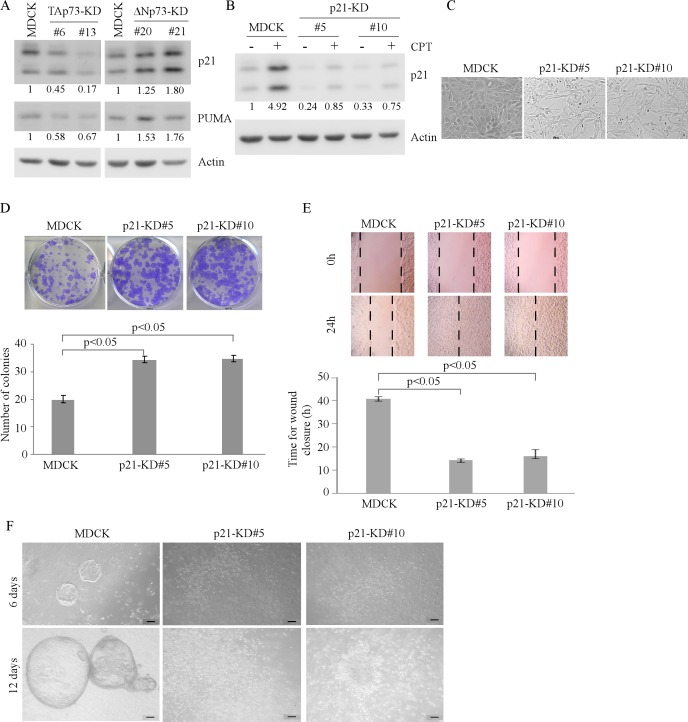
Knockdown of p21 alters MDCK cell morphogenesis in 2-D and 3-D cultures **A.** Knockdown of TAp73 decreases, whereas knockdown of ΔNp73 increases, the expression of p21 and PUMA. The levels of p21, PUMA, and actin were measured in parental MDCK, MDCK-TAp73-KD, and MDCK-ΔNp73-KD cells by Western blotting. **B.** Generation of MDCK cell lines in which p21 was stably knocked down. Parental MDCK and MDCK-p21-KD cells were mock-treated or treated with camptothecin for 18h and the level of p21 and actin protein was measured by Western blotting. **C.** Representative microscopic images of parental MDCK and MDCK-p21-KD cells in 2-D cultures. **D.** Top panel: colony formation assay was performed with parental MDCK or MDCK-p21-KD cells. Bottom panel: the number of colonies was counted and presented as Mean ± S.D. from three separate experiments. **E.** Wound healing assay was performed with parental MDCK and MDCK-p21-KD cells. Top panel: cell migration was determined by visual assessment of cells migrating into the wound for 24 h using a phase-contrast microscopy. The dashed lines indicate the wound edge. Bottom panel: the time required for would closure was measured and presented as Mean ± s.d. from three separate experiments. **F.** Representative images of parental MDCK and MDCK-p21-KD cells in 3-D culture for 6 d or 12 d. Scale bar: 50 μM.

Cell cycle arrest and apoptosis are known to be required for cyst formation in 3-D culture of MDCK cells [17]. Notably, we found that knockdown of TAp73 enhances whereas knockdown of ΔNp73 inhibits, MDCK cell proliferation (Figures 1D and 2C). These results prompted us to speculate that the enhanced cell proliferation leads to irregular cyst formation for MDCK-TAp73-KD cells in 3-D culture potentially via p21 and PUMA. Both p21 and PUMA are p53 family target genes, and are key regulators of growth inhibition. p21 mediates cell cycle arrest whereas PUMA mediates apoptosis [18, 19]. Indeed, we found that the level of p21 and PUMA was decreased in MDCK-TAp73-KD cells, but increased in MDCK-ΔNp73-KD cells (Figure 3A). To determine the role of p21 in cell morphogenesis, we generated stable MDCK cell lines in which p21 was knocked down. Two representative clones (clones #5 and #10) were shown in Figure 3B. As expected, the level of p21 was significantly reduced in MDCK-p21-KD cells as compared to that in parental MDCK cells regardless of camptothecin treatment. Interestingly, in 2-D culture, MDCK-p21-KD cells exhibited an elongated morphology (Figure 3C). These cells also proliferated and migrated faster than parental MDCK cells (Figure 3D-3E). Furthermore, MDCK-p21-KD cells were unable to form regular cyst structure in 3-D culture (Figure 3F).

**Figure 4:**
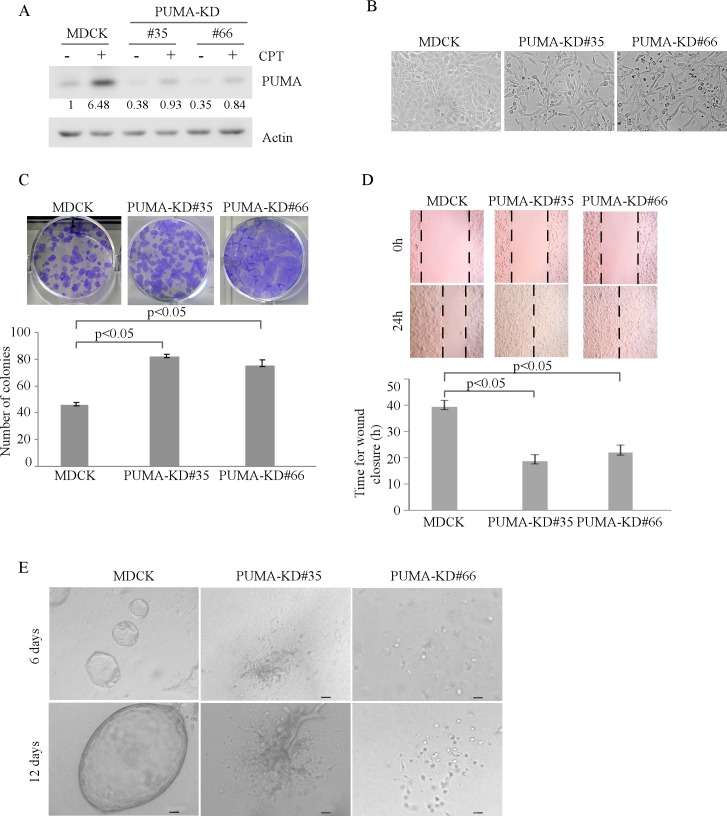
Knockdown of PUMA alters MDCK cell morphogenesis in 2-D and 3-D cultures **A.** Generation of MDCK cell lines in which PUMA was stably knocked down. Parental MDCK and MDCK-PUMA-KD cells were mock-treated or treated with camptothecin for 18h and the level of PUMA and actin protein was measured by Western blotting. **B.** Representative microscopic images of parental MDCK and MDCK-PUMA-KD cells in 2-D cultures. **C.** Top panel: colony formation assay was performed with parental MDCK or MDCK-PUMA-KD cells. Bottom panel: the number of colonies was counted and presented as Mean ± s.d. from three separate experiments. **D.** Wound healing assay was performed with parental MDCK and MDCK-PUMA-KD cells. Top panel: cell migration was determined by visual assessment of cells migrating into the wound for 24 h using a phase-contrast microscopy. The dashed lines indicate the wound edge. Bottom panel: the time required for would closure was measured and presented as Mean ± s.d. from three separate experiments. **E.** Representative images of parental MDCK and MDCK-PUMA-KD cells in 3-D culture for 6 d or 12 d. Scale bar: 50 μM.

Next, to determine the role of PUMA in regulating MDCK morphogenesis, we generated stable MDCK cell lines with PUMA knockdown. As shown in Figure 4A, the level of PUMA protein was markedly reduced in MDCK-PUMA-KD cells than that in parental MDCK cells. In addition, we found that like MDCK-p21-KD cells, MDCK-PUMA-KD cells displayed an elongated morphology in 2-D cultures (Figure 4B). Moreover, knockdown of PUMA increased MDCK cell proliferation and migration in 2-D culture (Figure 4C-4D), and disrupted cyst formation in 3-D culture (Figure 4E). Together, these data suggest that like TAp73, p21 and PUMA are required for MDCK cell morphogenesis in 2-D and 3-D cultures.

### TAp73, p21, PUMA, but not ΔNp73, are required for maintaining an appropriate level of EMT markers in MDCK cells

**Figure 5:**
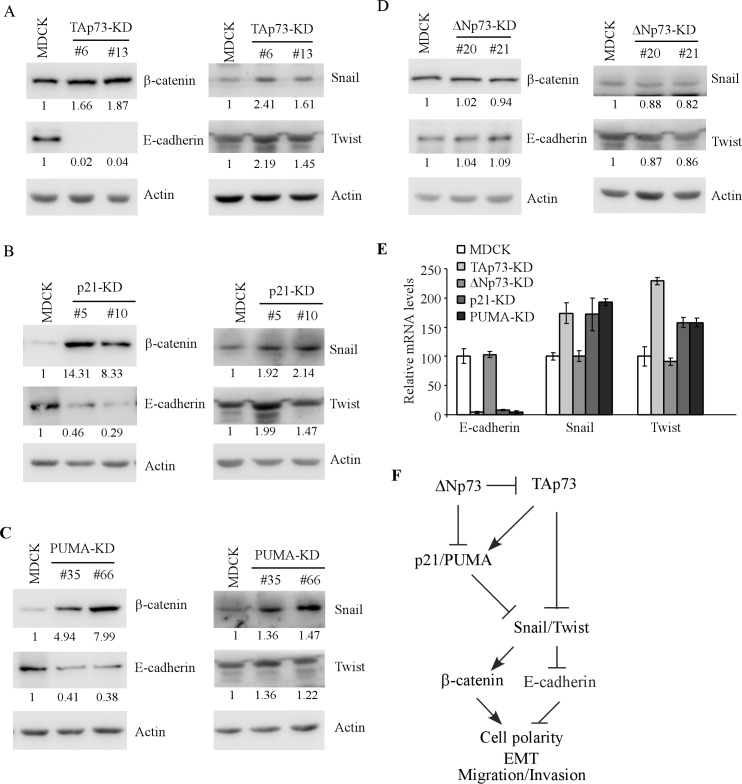
Knockdown of TAp73, p21, PUMA, but not ΔNp73, induces EMT phenotypes in MDCK cells **A.**-**D.** The levels of β-catenin, E-cadherin, Snail, Twist, and actin were determined by Western blotting with extracts from parental MDCK (A-D), MDCK-TAp73-KD **A.**, MDCK-p21-KD **B.**, MDCK-PUMA-KD **C.**, and MDCK-ΔNp73-KD **D.** cells. **E.** The levels of E-cadherin, Snail and Twist transcripts were measured by qRT-PCR in parental MDCK cells and MDCK cells with knockdown of TAp73, ΔNp73, p21 or PUMA. The level of Actin was measured as an internal control. **F.** A model for the role of p73, p21 and PUMA in MDCK cell morphogenesis.

Loss of epithelial cell morphology is often accompanied with EMT [20]. In addition, we noticed that MDCK cells deficient in TAp73, p21, or PUMA exhibited elongated, spindle-like morphology (Figures 1C, 3C, and 4B). Thus, the levels of EMT markers were examined in MDCK cells with knockdown of TAp73, ΔNp73, p21 or PUMA. We found that the level of β-catenin was increased whereas the level of E-cadherin was decreased in MDCK-TAp73-KD cells as compared to that in parental MDCK cells (Figure 5A, left panel). Consistent with this, both snail and Twist were increased in MDCK-TAp73-KD cells than that in parental MDCK cells (Figure 5A, right panel). Similarly, we found that knockdown of p21 or PUMA led to an increase in β-catenin, Snail, and Twist but a decrease in E-cadherin in MDCK cells (Figure 5B-5C). By contrast, knockdown of ΔNp73 in MDCK cells had little, if any, effect on the level of β-catenin, E-cadherin, Snail and Twist (Figure 5D). To confirm this, the level of E-cadherin, Snail, Twist transcripts, known to be regulated at the transcriptional level [21-24], was determined by qRT-PCR. We found that knockdown of TAp73, p21, or PUMA led to a significant reduction of E-cadherin transcript, but an increase in Snail and Twist transcripts (Figure 5E). By contrast, knockdown of ΔNp73 had no effect (Figure 5E). Together, these data suggest that knockdown of TAp73, p21, PUMA, but not ΔNp73, alters MDCK cell morphogenesis potentially via enhanced EMT.

## DISCUSSION

We showed previously that p63 and mutant p53 play a critical role in branching tubulogenesis [12, 13]. However, it is not clear whether p73 is involved in this process. In the current study, we explored the role of p73 in epithelial cell morphogenesis in MDCK cells. We found that TAp73, but not ΔNp73, is required for MDCK cell morphogenesis. Similarly, we found that both p21 and PUMA are required for MDCK cell morphogenesis. Furthermore, we showed that MDCK cells deficient in TAp73, p21, or PUMA exhibit an elongated morphology and induce EMT phenotypes. Together, our results suggest that TAp73 and its downstream targets, p21 and PUMA, are required for MDCK cell morphogenesis by maintaining an appropriate level of EMT. A model for the role of p73, p21 and PUMA in MDCK morphogenesis is proposed in Figure 5F.

EMT is a phenotype alteration that occurs in tumorigenesis and tumor progression. It is characterized by alteration of gene expression, leading to reduced intercellular adhesion and increased cell motility and invasiveness. Tubular EMT is a progress in which renal tubular cells lose their epithelial phenotype and acquire new features of mesenchyme. In this study, we found that knockdown of TAp73 but not ΔNp73 induces EMT phenotypes in MDCK cells with a decreased expression of E-cadherin and an increased expression of β-catenin, Snail-1 and Twist (Figure 5A and 5D-5E). Consistent with this, TAp73, but not ΔNp73, is found to be required for maintaining MCF10A cell polarity and morphogenesis via suppressing EMT [25]. As EMT induction is often associated with cancer cell invasive and metastatic properties [26], thus, restoration of TAp73 expression to suppress EMT program may be a valuable approach for tumor suppression.

In our study, we found that knockdown of TAp73 markedly alters MDCK cell morphology in 2-D and 3-D cultures (Figure 1). In contrast, knockdown of ΔNp73 inhibits cell proliferation and migration in 2-D culture, and delays but not disrupts cyst formation on 3-D culture (Figure 2). These results suggest that an appropriate level of EMT is critical for MDCK cell morphogenesis in 3-D culture. Interestingly, we also showed that TAp63, but not ΔNp63, is required for MDCK cyst formation [13]. However, down-regulation of p53 alone is not sufficient to alter tubular formation in 3-D culture, but promotes cell proliferation and migration through loss of induction of growth-suppressing genes [13]. These results suggest that various p53 family members may exert a tissue-specific function during development. These studies also provide evidence that TAp73 may cooperate with other p53 family proteins in cell morphogenesis. Indeed, p73 and p53 cooperates or compensates for each other for kidney development [27]. Thus, it would be interesting to further characterize how the p53 family proteins cooperate to modulate the EMT program and consequently, affect the branching morphogenesis.

The finding that both p21 and PUMA are required for cyst formation (Figures 3-4) suggests that both cell cycle arrest and apoptosis are critical for tubular formation. Interestingly, we also found that both p21 and PUMA are required for inner cell clearance during lumen formation in MCF10A cells [28]. Notably, knockdown of p21 or PUMA only modestly alters MCF10A cell morphogenesis [28]. However, knockdown of p21 or PUMA leads to severe morphological alteration in MDCK cells (Figures 3F and 4E). These results suggest that growth suppression mediated by p21 or PUMA are crucial for MDCK cell cyst formation in 3-D culture. However, in MCF10A cells, other regulators, such as Bim, are also required for the clearance of inner and outer cells during acinar formation [29]. Finally, since p21 and PUMA are targets of TAp63 and TAp73, it would be interesting to determine how TAp73 and TAp63 cooperate with p21 and PUMA to modulate epithelial cell morphogenesis.

## MATERIALS AND METHODS

### Reagents

Bovine collagen solution (3.2 mg/mL) was purchased from Advanced Biomatrix (Poway, CA). MEM medium and non-essential amino acid were purchased from Invitrogen (Carlsbad, CA).

### Plasmid construction and cell line generation

To generate vectors expressing shRNAs against canine TAp73, ΔNp73, p21, or PUMA under the control of the U6 promoter, two 62-base oligos were annealed and then cloned into pBabe-U6 shRNA expression vector. The resulting plasmids were designed as pBabe-U6-shTAp73, pBabe-U6-shΔNp73, pBabe-U6-shP21, and pBabe-U6-shPUMA. The shRNA oligos with the siRNA targeting region shown in uppercase are listed in Table 1. Knockdown cell lines were selected with puromycin and confirmed by RT-PCR and/or Western blot analysis.

**Table 1:** The oligos used for generation of shRNA expression vectors

siTAp73-1	tcgaggtcc GCTCCACCTTTGACACCAT ttcaagaga ATGGTGTCAAAGGTGGAGC tttttg gatccaaaaa GCTCCACCTTTGACACCAT tctcttgaa ATGGTGTCAAAGGTGGAGC ggacc
siTAp73-2	tcgaggtcc GCCAAACCAGGGAAACAGT ttcaagaga ACTGTTTCCCTGGTTTGGC tttttg gatccaaaaa GCCAAACCAGGGAAACAGT tctcttgaa ACTGTTTCCCTGGTTTGGC ggacc
siΔNp73-1	tcgaggtcc GAGCTGTGTGCTTTCACAT ttcaagaga ATGTGAAAGCACACAGCTC tttttg gatccaaaaa GAGCTGTGTGCTTTCACAT tctcttgaa ATGTGAAAGCACACAGCTC ggacc
siΔNp73-1	tcgaggtcc CCTCTAGAATCCAGCAGCT ttcaagaga AGCTGCTGGATTCTAGAGG tttttg gatccaaaaa CCTCTAGAATCCAGCAGCT tctcttgaa AGCTGCTGGATTCTAGAGG ggacc
sip21-1	tcgaggtcc GCGATGGAACTTTGACTTC ttcaagaga GAAGTCAAAGTTCCATCGC tttttg gatccaaaaa GCGATGGAACTTTGACTTC tctcttgaa GAAGTCAAAGTTCCATCGC ggacc
sip21-1	tcgaggtcc GGCAGACCAGCATGACAGA ttcaagaga TCTGTCATGCTGGTCTGCC tttttg gatccaaaaa GGCAGACCAGCATGACAGA tctcttgaa TCTGTCATGCTGGTCTGCC ggacc
siPUMA-1	tcgaggtcc GGGTCCTGTACAATCTCAT ttcaagaga ATGAGATTGTACAGGACCC tttttg gatccaaaaa GGGTCCTGTACAATCTCAT tctcttgaa ATGAGATTGTACAGGACCC ggacc
siPUMA-2	tcgaggtcc GGAGATGGAGCCCAATTAG ttcaagaga CTAATTGGGCTCCATCTCC tttttg gatccaaaaa GGAGATGGAGCCCAATTAG tctcttgaa CTAATTGGGCTCCATCTCC ggacc

### Cell culture

The MDCK cell line was obtained from American Type Culture Collection (ATCC, Manassas, VA) and cultured in MEM medium supplemented with 10% fetal bovine serum and 1% non-essential amino acid. The overlay 3-D culture was carried out as described previously with some modifications [12]. Briefly, 12-well culture plates were pre-coated evenly with 1.0 mg/mL pre-mixed collagen gel and then incubated at 37 °C for 30 min to allow the collagen gel to solidify. MDCK cells or MDCK cells with TAp73-KD, ΔNp73-KD, p21-KD or PUMA-KD (5,000 cells) suspended in 1.0 mL collagen gel mixture were seeded on the top of pre-gelled layer, and then incubated for 30 min at 37 °C to solidify. MEM growth medium was gently added to the top of each gel and incubated at 37 °C in a humidified 5% CO_2_. Culture medium was renewed every third day.

### RNA isolation, RT-PCR and qRT–PCR

Total RNA was extracted from cells using TRIzol (Invitrogen Life Technoloogies, Grand Island, NY) according to the manufacturer's instructions. cDNA was synthesized using M-MLV Reverse Transcriptase Kit (Promega Corporation, Madison, WI) according to the manufacturer's protocol. Quantitative PCR was performed in 20 μL reaction using 2x IQ SYBR Green Supermix (Bio-Rad) and 5μM primers. Reactions were run on a Real-time platform (Eppendorf *Mastercycler epRealpex*^2^, Germany) using a three-step cycling program: 95°C for 15 min, followed by 40 cycles of 95°C for 15 s, 60°C for 30 s, and 68°C for 30 s. A melting curve (57-95°C) was generated at the end of each run to verify the specificity. The primers for TAp73, ΔNp73, Actin, E-cadherin, Snail and Twist are listed in Table 2.

**Table 2:** The primers used by RT-PCR

ΔNp73	Sense: 5′-acctcttgtaggccctcctg-3′ Antisense: 5′-ccttgcatgtgaaagcaca-3′
TAp73	Sense: 5′-cagggaaacagtgaggtggt-3′ Antisense: 5′-gaaggtgacgtcgaagtggt-3′
Snail-1	Sense: 5′-cggctccctcgtccttctcttccac-3′ Antisense: 5′-ccccctgagcagcctgattcttggt-3′
Twist	Sense: 5′-ggctccagctcgcccgtgtcccccg-3′ Antisense: 5′-cgctagtgggaggcggacatggacc-3′
E-cadherin	Sense: 5′-ggtgctcacatttcccagtt-3′ Antisense: 5′-tccagaggctcagtcacctt-3′
Actin	Sense: 5′-ctgaagtaccccatcgagcacggca-3′ Antisense: 5′-ggatagcacagcctggatagcaacg-3′

### Western blot analysis

Western blotting was performed as described [30]. Antibodies used were purchased from Bethyl (anti-p73), ProSci (anti-PUMA), Santa Cruz Biotechnology (anti-p21 (H164), anti-β-catenin (E-5), anti-Snail-1, anti-Twist), BD Transduction Laboratories (anti-E-cadherin, San Jose, CA), Sigma (anti-actin, St. Louis, MO), and BioRad (secondary antibodies against rabbit or mouse IgG conjugated with HRP, Life Science Research, Hercules, CA). Experiments were repeated at least three times.

### Colony formation assay

MDCK cells were cultured in a 6-well plate for ~12 d and then fixed with methanol/glacial acetic acid (7:1) followed by staining with 0.1% crystal violet. Experiments were conducted in triplicate.

### Wound healing assay

Cells were grown in a 6-well plate for 24 h. The monolayers were wounded by scraping with a P200 micropipette tip and washed two times with PBS. At specified time points after the scraping, cell migration was captured using phase contrast microscopy and cell monolayers were photographed using a Leica Wetzlar microscope. Migration rate of cells was measured by averaging the time required to close the borders of cells. At least six regions were analyzed in each well, and the result was expressed as the mean ± s.d.

### Statistical analysis

Data were presented as Mean ± s.d. Statistical significance was determined by Student's *t* test. Values of *P* < 0.05 were considered significant.

## References

[1] Conforti F. (2012). Regulation of p73 activity by post-translational modifications. Cell Death Dis.

[2] Jost C.A., Marin M.C., Kaelin W.G. (1997). p73 is a simian [correction of human] p53-related protein that can induce apoptosis. Nature.

[3] Tomasini R. (2008). TAp73 knockout shows genomic instability with infertility and tumor suppressor functions. Genes Dev.

[4] Rufini A. (2012). TAp73 depletion accelerates aging through metabolic dysregulation. Genes Dev.

[5] Tissir F. (2009). DeltaNp73 regulates neuronal survival in vivo. Proc Natl Acad Sci U S A.

[6] Wilhelm M.T. (2010). Isoform-specific p73 knockout mice reveal a novel role for delta Np73 in the DNA damage response pathway. Genes Dev.

[7] Bacallao R., Fine L.G. (1989). Molecular events in the organization of renal tubular epithelium: from nephrogenesis to regeneration. Am J Physiol.

[8] Liu Y. (2004). Epithelial to mesenchymal transition in renal fibrogenesis: pathologic significance, molecular mechanism. J Am Soc Nephrol.

[9] McAteer J.A., Evan A.P., Gardner K.D. (1987). Morphogenetic clonal growth of kidney epithelial cell line MDCK. Anat Rec.

[10] Saxen L., Sariola H (1987). Early organogenesis of the kidney. Pediatr Nephrol.

[11] Elia N., Lippincott-Schwartz J (2009). Culturing MDCK cells in three dimensions for analyzing intracellular dynamics. Curr Protoc Cell Biol.

[12] Zhang Y., Yan W., Chen X (2013). Mutant p53 cooperates with knockdown of endogenous wild-type p53 to disrupt tubulogenesis in Madin-Darby canine kidney cells. PLoS One.

[13] Zhang Y., Yan W., Chen X (2014). P63 regulates tubular formation via epithelial-to-mesenchymal transition. Oncogene.

[14] Jung Y.S., Qian Y., Chen X (2011). The p73 tumor suppressor is targeted by Pirh2 RING finger E3 ubiquitin ligase for the proteasome-dependent degradation. J Biol Chem.

[15] Urist M. (2004). p73 induction after DNA damage is regulated by checkpoint kinases Chk1 and Chk2. Genes Dev.

[16] Maisse C. (2004). DNA damage induces the rapid and selective degradation of the DeltaNp73 isoform, allowing apoptosis to occur. Cell Death Differ.

[17] Bryant D.M., Mostov K.E. (2008). From cells to organs: building polarized tissue. Nat Rev Mol Cell Biol.

[18] el-Deiry W.S. (1993). WAF1, a potential mediator of p53 tumor suppression. Cell.

[19] Han J. (2001). Expression of bbc3, a pro-apoptotic BH3-only gene, is regulated by diverse cell death and survival signals. Proc Natl Acad Sci U S A.

[20] Christiansen J.J., Rajasekaran A.K. (2006). Reassessing epithelial to mesenchymal transition as a prerequisite for carcinoma invasion and metastasis. Cancer Res.

[21] Moody S.E. (2005). The transcriptional repressor Snail promotes mammary tumor recurrence. Cancer Cell.

[22] Tan E.J. (2012). Regulation of transcription factor Twist expression by the DNA architectural protein high mobility group A2 during epithelial-to-mesenchymal transition. J Biol Chem.

[23] Thuault S. (2008). HMGA2 and Smads co-regulate SNAIL1 expression during induction of epithelial-to-mesenchymal transition. J Biol Chem.

[24] Thuault S. (2006). Transforming growth factor-beta employs HMGA2 to elicit epithelial-mesenchymal transition. J Cell Biol.

[25] Zhang Y. (2012). Mammary epithelial cell polarity is regulated differentially by p73 isoforms via epithelial-to-mesenchymal transition. J Biol Chem.

[26] Singh A., Settleman J (2010). EMT, cancer stem cells and drug resistance: an emerging axis of evil in the war on cancer. Oncogene.

[27] Saifudeen Z. (2005). Spatiotemporal switch from DeltaNp73 to TAp73 isoforms during nephrogenesis: impact on differentiation gene expression. J Biol Chem.

[28] Zhang Y. (2013). PUMA Cooperates with p21 to Regulate Mammary Epithelial Morphogenesis and Epithelial-To-Mesenchymal Transition. PLoS One.

[29] Reginato M.J. (2005). Bim regulation of lumen formation in cultured mammary epithelial acini is targeted by oncogenes. Mol Cell Biol.

[30] Zhang Y., Yan W., Chen X (2011). Mutant p53 disrupts MCF-10A cell polarity in three-dimensional culture via epithelial-to-mesenchymal transitions. J Biol Chem.

